# Crosstalk between long noncoding RNA and microRNA in Cancer

**DOI:** 10.1007/s13402-023-00806-9

**Published:** 2023-05-28

**Authors:** Rahul Bhattacharjee, Neeraj Prabhakar, Lamha Kumar, Arkadyuti Bhattacharjee, Sulagna Kar, Sumira Malik, Dhruv Kumar, Janne Ruokolainen, Arvind Negi, Niraj Kumar Jha, Kavindra Kumar Kesari

**Affiliations:** 1 KIIT School of Biotechnology, Kalinga Institute of Industrial Technology (KIIT-DU), Bhubaneswar, Odisha India; 2 Present Address: Centre for Structural System Biology, Department of Physics, University of Hamburg, c/o DESY, Building 15, Notkestr. 852267, Hamburg, Germany; 3 School of Biology, Indian Institute of Science Education and Research, Thiruvananthapuram, India; 4 Amity Institute of Biotechnology, Amity University Jharkhand, Ranchi, Jharkhand, 834001 India; 5 School of Health Sciences and Technology (SoHST), UPES University, Dehradun, Uttarakhand India; 6 Department of Applied Physics, School of Science, Aalto University, Espoo, 00076 Finland; 7 Department of Bioproducts and Biosystems, School of Chemical Engineering, Aalto University, Espoo, 00076 Finland; 8 Department of Biotechnology, School of Engineering and Technology (SET), Sharda University, Greater Noida, 201310 UP India; 9 School of Bioengineering & Biosciences, Lovely Professional University, Phagwara, 144411 India; 10 Department of Biotechnology, School of Applied & Life Sciences (SALS), Uttaranchal University, Dehradun, 248007 India; 11 Faculty of Biological and Environmental Sciences, University of Helsinki, Biocentre 3, Helsinki, Finland; 12 Pharmacy, Abo Akademi University, Tykistökatu 6A, Turku, Finland

**Keywords:** MicroRNA, Long-noncoding RNA, Cancer, Epithelial-mesenchymal transition, Metastasis, Angiogenesis, Neovascularization, Vascular mimicry

## Abstract

miRNAs and lncRNAs play a central role in cancer-associated gene regulations. The dysregulated expression of lncRNAs has been reported as a hallmark of cancer progression, acting as an independent prediction marker for an individual cancer patient. The interplay of miRNA and lncRNA decides the variation of tumorigenesis that could be mediated by acting as sponges for endogenous RNAs, regulating miRNA decay, mediating intra-chromosomal interactions, and modulating epigenetic components. This paper focuses on the influence of crosstalk between lncRNA and miRNA on cancer hallmarks such as epithelial-mesenchymal transition, hijacking cell death, metastasis, and invasion. Other cellular roles of crosstalks, such as neovascularization, vascular mimicry, and angiogenesis were also discussed. Additionally, we reviewed crosstalk mechanism with specific host immune responses and targeting interplay (between lncRNA and miRNA) in cancer diagnosis and management.

**Graphic Abstract:**

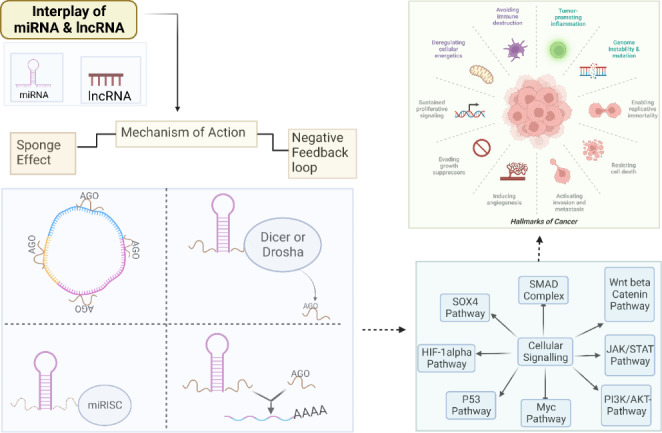

**Study Highlights**:

- A negative regulatory or sponging mechanism between lncRNA and miRNA has been discussed as a possible contributor to cancer aetiology.
- This review explains how miRNA and lncRNA crosstalk affects cancer hallmarks and tumour features and how it can be used to treat carcinogenesis.
- This review may enable the development of new immunotherapies or inhibitors targeting cell signaling or molecular effectors of miRNA-lncRNA crosstalk.

## Introduction

Cancer is a multifactorial disease caused by the existence of driver mutations that cause proto-oncogene to be activated and inactivation of tumor suppressor genes. Such events cause a change in cell function, characterized as cancer hallmarks, and ultimately aggravates the chemoresistance to conventional therapies [1–6]. Apart from gene mutations, the expression of noncoding RNAs (ncRNAs) such as micro-RNAs (miRNAs) and long noncoding RNAs (lncRNAs) has been described [7, 8]. miRNAs are single-stranded RNAs constituting 18–25 nucleotides that play a significant role in biological processes, such as stem cell differentiation, development, and tissue identity *via* negatively regulating the mRNA transcripts [9, 10]. LncRNAs consisting of more than 200 nucleotides. They may interact with nucleic acids and proteins, controlling a range of cellular functions, including post-transcriptional mRNA regulation, scaffolding, post-transcriptional modification, transcription, and chromatin modification [11, 12]. In addition, miRNAs and lncRNAs play a central role in several gene regulations [13]. lncRNAs behave as molecular decoys by capturing miRNAs and preventing them from interacting with their respective messenger RNAs (mRNA) [12, 14] for their crosstalk with miRNAs to control several biological activities [15].

The dysregulated expression of lncRNAs has been reported as a hallmark of cancer progression, acting as an independent prediction marker for an individual cancer patient [16, 17]. A recent study reported a significant functional contribution of lncRNAs at transcriptional levels, targeting *cis*-acting regulatory sites or *trans*-regulatory sites. Through these mechanistic approaches, lncRNAs are expected to cause cancer progression. Therefore, they serve as potential targets to study cancer progression as their dysregulated pattern is distinctive depending on specific tumorigenic cells and tissues [18]. The interplay of miRNA and lncRNA decides the variation of tumorigenesis that could be mediated by acting as sponges for endogenous RNAs, regulating miRNA decay, mediating intra-chromosomal interactions, and modulating epigenetic components [19].

Competitive endogenous RNAs (ceRNAs) are transcripts that have been shown to compete for shared miRNA sites at the post-transcriptional level, making them ideal indicators for cancer progression [20]. The lncRNA possesses numerous miRNA response elements MREs sites, eventually leading to their interaction with more miRNAs due to the large number of MREs. The recent studies reported the remarkable effect of lncRNA-BGL3 acting as ceRNA for various miRs such as miR-17, -20a/-20b, -93, miR-106a/-106b in inhibiting repression of phosphatase and tensin homolog mRNAs causing physiological and pathological conditions. Oncocers are ceRNAs (Competing endogenous RNAs) that play essential roles in oncogenic pathways, and their crosstalk is studied by sponging miRNAs in these pathways [21].

To an extent, the role of miRNAs or lncRNAs in multiple cancers has been understood at the molecular level but requires additional studies to gain more insights into their differential cellular functions. The role of specific miRNA patterns has been studied for therapeutic intervention [22]. The role of lncRNAs in malignant cancers has recently drawn a lot of attention [23–26]. There is also a growing recognition of regulatory RNA crosstalk as a result of lncRNA-miRNA interactions, which affect many events in carcinogenesis and metastasis. A transcriptome-wide analysis of the interactions between lncRNA and miRNA resulted in the creation of a genome-wide map displaying these interactions [27]. The field of lncRNA-miRNA interaction is still nascent, and the understanding of specific regulation is still emerging. It will take time for the global research community to offer more functional data that will allow a choice of therapeutic intervention targeting lncRNA-miRNA in cancer [28].

**Fig. 1:**
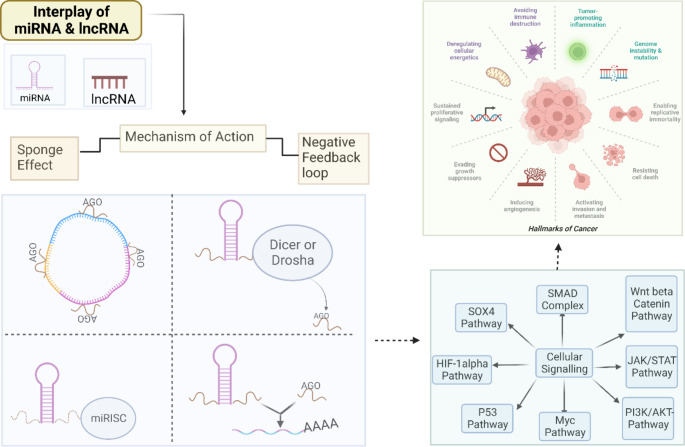
Landscaping of miRNA-lncRNA interaction in cancer

This review may interest global research community to study cancer by focusing on lncRNA-miRNA interactions rather than only miRNA or lncRNA (Fig. 1). We have also covered the potential mechanism of action and influence of miRNA and lncRNA interactions on cancer hallmarks.

## Mechanism of the interplay between miRNA and lncRNA

**Fig. 2:**
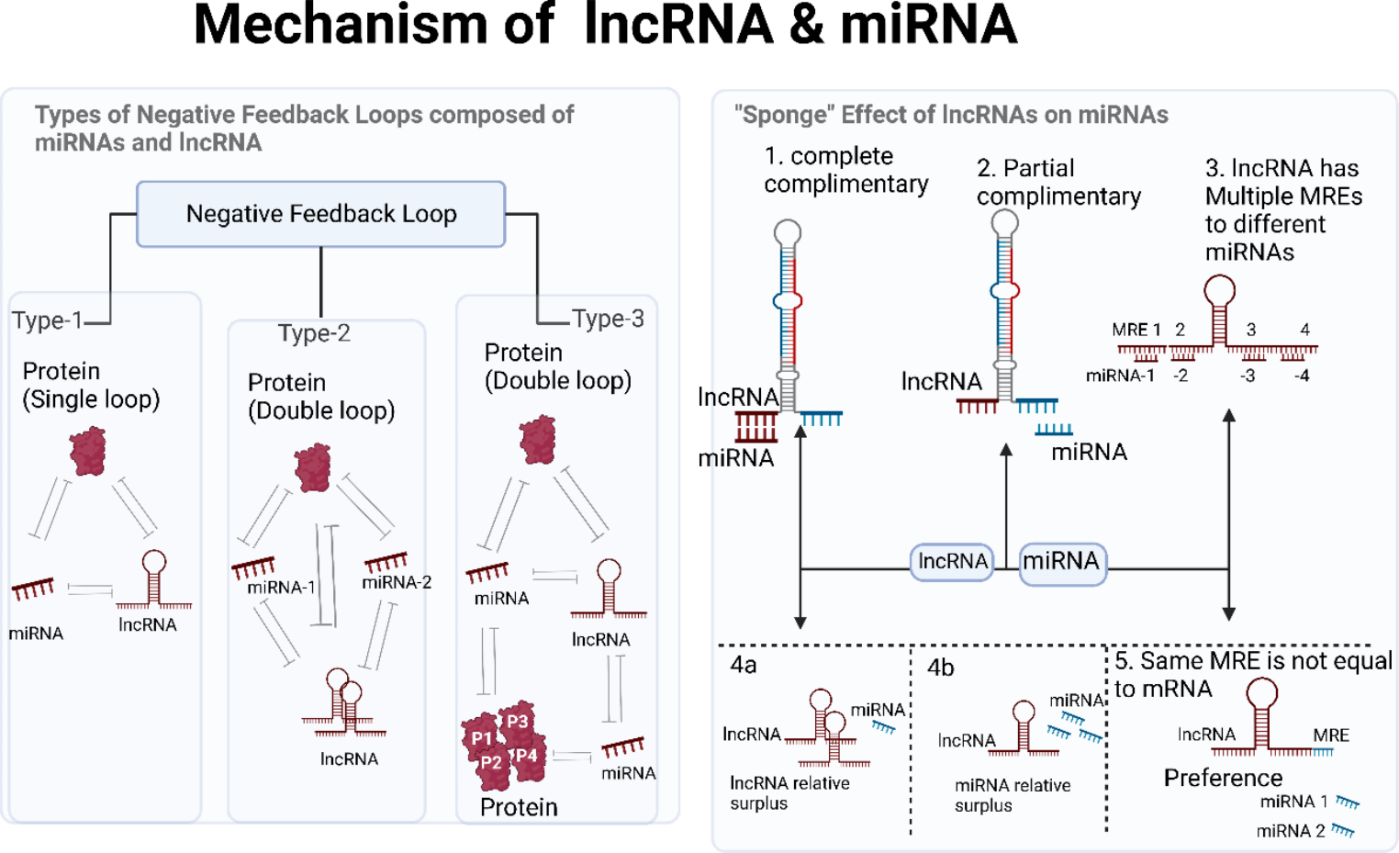
Schematic representation of the crosstalk mechanism between miRNA and lncRNA *via* negative regulation (a) and sponging effect (b)

LncRNAs regulate gene expression at multiple levels (DNA, RNA, and proteins) by modulating chromatin structure, post-translational modification, RNA modification, and stability. We have focused on explaining the two functioning processes that are responsible for the interaction between lncRNA and miRNA (Fig. 2).

### Sponge effect of lncRNAs on miRNAs

LncRNAs and miRNAs play essential roles in genetic control. Unfortunately, many of these transcripts’ roles are yet unknown [29]. Substantial crosstalk between miRNAs and lncRNAs results in binding competition between the miRNA and lncRNA *via* targets for regulating multiple proteins and transcription factors [30]. In the case of ceRNAs, a lncRNA performs its job by sequestering miRNAs, hence decreasing their levels. As a result, miRNAs are less likely to bind to their mRNA targets, a process known as the sponge effect. Predicting miRNA targets in mRNAs is possible using a variety of techniques. Sponge ceRNAs are lncRNAs, yet few methods exist for predicting miRNA response elements (MREs) in these RNAs [31–33]. Hence, the sponge effect of MRE-lncRNA crosstalk irreplaceably impacts diverse cancer progressions. Crosstalk and communication between lncRNAs with several common MREs play a significant role in biochemical events at the cellular and molecular level, causing distinct physiological and pathological states. The positionally selective phenomenon of lncRNA BC032469 targeting miR-1207-5p rather than its corresponding miR-1266 seed region inducing gene downregulation was recently reported. This shows that lncRNAs target their core miRNA sites while leaving others unaffected. Sponging effects mediate lncRNA and miRNA biochemical regulation in cancer progression [34].

The above-mentioned sponging activity could be described by explaining the two kinds of crosstalk between lncRNAs and miRNAs, which involve either complete or partial complementary modes [34]. MREs contain conserved target sites and mediate the mechanism by which miRNA typically attaches to a partially complementary target gene sequence. lncRNAs regulate miRNA MREs and play an important role in post-transcriptional regulation [35]. The sponging impact of miRNA and lncRNA involves lncRNA, which is adversely controlled by miRNA. The mature lncRNA incomplete complementation has a hat-like structure with a poly5-A tail at the end, a 5’UTR, a 3’UTR and multiple MREs, connecting with miRNAs. The more lncRNAs share multiple MREs, the crosstalk will proceed successfully [21].

Some lncRNAs without MRE use the sponging effect with the reciprocal miRNA to express a superior selection when there are several miRNAs present. Similarly, in the instance of lncRNA BC032469, which possessed elements complementary to miR-1207-5p and miR-1266 in the seed regions but no MRE, weakening the down-regulation of miR-1207-5p-dependent target genes [36]. MREs in lncRNAs express a position preference in mid-region and at the 3’ends of the lncRNAs when it comes to the AGO binding sites, regulatory elements harbor a particular pattern crosswise to the transcripts [37]. Furthermore, the sponging effect’s influence is dependent on a specific spatial-temporal distribution [38]. The lncRNA UCA1 is one such case where it interacts with miR-184 *via* a sponging mechanism for causing cellular proliferation for prostate cancer [39, 40]. In summary, the lncRNA may compete for miRNA MREs, reducing miRNA repression of target genes, so indirectly affecting the expression levels of target genes and ultimately aiding in cancer regulation. miRNAs and lncRNAs constitute a negative feedback loop.

### 2.2. Negative feedback loop:

The capacity to produce a negative feedback regulation pathway for the crosstalk of lncRNA and miRNA is an additional mechanism critical for a functional miRNA and lncRNAs interaction. Both miRNAs and lncRNAs play roles in regulating gene expression. The expression of lncRNAs is indirectly influenced by miRNAs. In a manner analogous to an enhancer function, miRNA and lncRNA interactions generate regulatory network transcriptomes that affect the expression of nearby genes [41]. By inhibiting DNA methylase, the DLK1-MEG3 imprinted domain, which contains the tumor suppressor factor MEG3 lncRNA, promotes cancer growth [42]. MiRNAs degrade lncRNAs in an AGO-dependent way by binding to target lncRNA 3’UTR inside the RISC, causing complete mRNA destruction or ribosomal machinery blockage for gene silencing in tumors [34, 43].

Another negative feedback loop mechanism is the lncRNA-mediated downregulation of miRNA expression. Although, there are variations in sequence between lncRNAs and miRNAs, the lncRNAs could be used as miRNA precursors to alter miRNA regulation [30]. Although, H19 has a G as its major base and miR675 has either a G or a C, their interaction is typical of the ncRNA family, which includes members with widely varying degrees of nucleotide conservation and mutation rates. Inverted regions in the mature miRNAs ensure the stability of the stem-loop structure in pre-miRNAs. The integrity of the stem-loop structure of pre-miRNA is ensured by its structure. Due to the nucleotide makeup, different lengths are required for optimal functioning. Additionally, lncRNAs compete with miRNA targets (particular mRNAs), and there is negative regulation of miRNA for miRNA targets by lncRNAs, which compete with the target 3’UTR of the mRNA [32, 44]. In addition, nanog silencing in breast cancer is caused by the interaction of lncRNA FEZF1-AS1, which blocks miRNA30a, competitively [45]. In addition, lncRNA H19 binds to proteins in the miRNA production regulatory complex, PCAF/hnRNPU/Pol RNA II, to boost histone acetylation upstream of miR200 [46].

Overexpression of lncRNAs such as miR100HG, miR100, and miR125b is associated with resistance to the anti-cancer drug cetuximab in several cancer types. Coordination between miR100 and miR125b inhibits five Wnt-beta catenin negative regulators for mitochondrial genome preservation via a negative feedback loop involving MIR100HG and TF GATA binding protein 6 (GATA6). Using miR125b as a target, GATA6 suppresses MIR100HG, which in turn suppresses Wnt signaling through miR125b suppression and restores cetuximab sensitivity in cells that have been resistant to the drug [47]. Furthermore, lncRNA UCA1 interacts with miR-204 via a negative impacting mechanism, resulting in glioma progression by glioma cell migration and proliferation via downregulation of anti-miR-182 protein [48].

UCA1, a lncRNA involved in cellular proliferation and migration, interacts with miR-28-5p to upregulate homeobox B3 and cause angiogenesis in colon cancer [49]. Additionally, the lncRNA UCA1 promotes cellular proliferation and migration in a bromodomain 4, c-Myc, and growth factor-binding protein 5-dependent way [50–52] after interacting with miRNA 135 in a competitive method. Similar research also discovered that EZH2 cooperates with miR-26a-2 and miR-101 to suppress c-Myc and HIF-1a/1b. It has been found that EZH2 collaborates with miR-26a-2 and miR-101 to negatively regulate c-Myc and HIF-1a/1b [53, 54]. Moreover, a negative feedback loop involving miRNA-101, miR-26a, and EZH2 inhibits cell cycle regulatory factors [53–55].

By further chromatin changes, the lncRNAs affect miRNA expression [56]. This process underlies the crosstalk between microRNAs and lncRNAs in hepatocellular carcinoma, where overexpression of histone deacetylase-4 (HDAC4) inhibits miRNA-200a and vice versa [57].

## Ultra-conserved regions and crosstalk of miRNA and lncRNA

A significant proportion of transcripts produced by these UCRs (Ultraconserved regions) have secondary RNA structures and are found in clusters with other functional noncoding elements [58]. B-cell lymphocytes have the greatest number of transcribed UCRs, and various UCR signatures have been associated to human carcinomas and leukemia. The expression of nineteen UCRs, eight raised and eleven decreased, differed in CLL versus normal hematological tissues [58, 59]. The cancer-associated genomic region (CAGR) comprises a cluster of 7 UCRs (uc. 347 to uc. 353), two of which are transcribed UCRs (T-UCRs) expressed differentially between malignant and normal B-CLL CD5 + cells [58]. Additionally, there is a relationship between the expression of ZAP-70, a well-established prognostic marker for CLL, and a signature of five T-UCRs, including uc. 348 A(P) (N), uc. 346 A(P), uc. 215(N), uc. 160(N), and 269 A. (N). The levels of expression of the five T-UCRs mentioned above were discovered to be negatively linked with the expression profile of CLL-specific miRNAs [59].

As a result, lncRNAs and small RNA biology are intricately intertwined; many lncRNAs appear to function as precursors for small RNA species, particularly miRNAs. The possibility of complex regulation processes involving long noncoding RNAs and short RNAs, meaning that the two types of noncoding RNAs may interact. Given the increasing importance of lncRNAs in biomolecular regulatory interactions within cells and their role in the etiology of human disease and cancer, a thorough examination of their functions is required to shed light on the specific pathways and regulatory circuits in which they are involved [59]. Notwithstanding the fact that certain research suggests a link between UCRs and small RNA biology. A slew more investigations are needed to establish a clear link between UCRs and the crosstalk of lncRNAs and miRNAs in cancer.

## Impact of the interplay of miRNA and lncRNA

Several hallmarks, tumor features, and the tumor microenvironment (TME) are modulated by the crosstalk of miRNA and lncRNA, ultimately leading to progression. The tumor growth of several cancer pathways relies heavily on the regulatory network of the interplay between lncRNA and miRNA. This review explains how miRNA and lncRNA crosstalk can lead to immunomodulation, which can cause cancer development via angiogenesis, neovascularization, Epithelial-Mesenchymal Transition (EMT), Vascular Mimicry (VM), suppression of apoptosis, invasion, and metastasis.

### Angiogenesis and interplay of miRNA and lncRNA

**Fig. 3:**
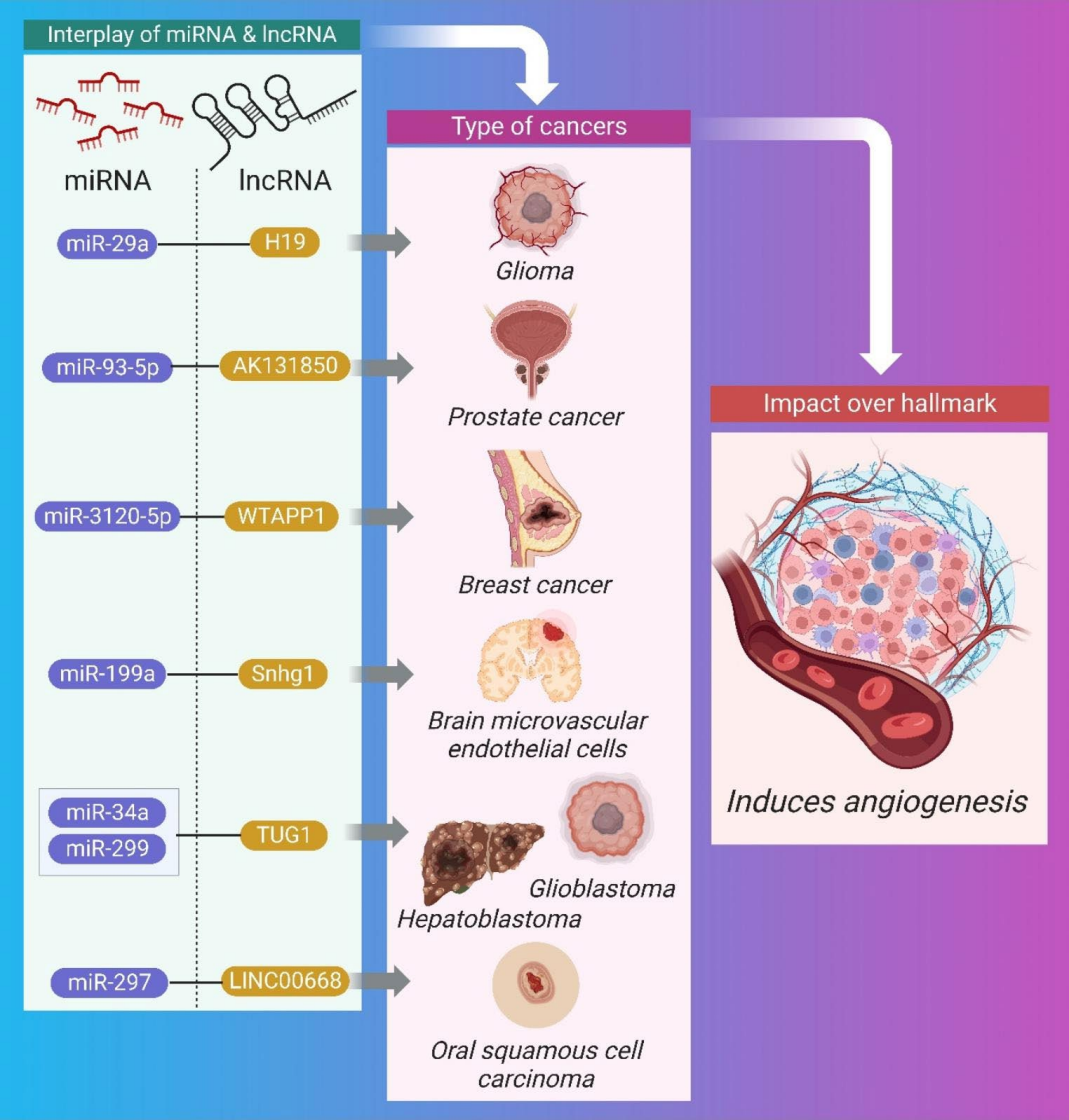
Schematic Representation of the interplay between miRNA and lncRNA causing brain cell carcinoma, breast cancer, oral cancer, and glioma through angiogenesis

The process by which pre-existing blood vessels are used to form capillary networks because of the requirement for nutrients in specific tissue regions is called angiogenesis [60]. It is one of the most critical aspects of all cancer processes, and it is closely regulated by a network of angiogenesis inhibitors (such as VASH2) and activators (such as VEGF) [61]. Tumor cells need resources, therefore, they manipulate angiogenesis to their benefit, causing the delicate activator-inhibitor balance to shift [62]. Therapeutic strategies targeting the crosstalk between miRNA and lncRNA, or the development of inhibitors against the downstream effector molecule that causes angiogenesis upon crosstalk, require a thorough understanding of the regulatory network and role of angiogenesis. Figure 3 illustrates the impact of crosstalk between miRNA and lncRNA via angiogenesis and is summarized in Table 1.

**Table 1:** Synopsis of the Crosstalk between miRNA and lncRNA for angiogenesis

Cancer	Pre-clinical	Mechanism	miRNA	lncRNA	Impact over Hallmark	Therapeutic intervention for tumor regression	Cellular Signalling/ Molecular effectors	Ref.
Glioblastoma	in vivo (human)	Sponging	miRNA-299	TUG1	Induces angiogenesis, Tumor regression	TUG1 knockout inhibited glioma	VEGF-A	[63]
Oral squamous cell	in vitro	Sponging	miR-297	LINC00668	increased cell proliferation via angiogenesis, Tumor regression	LINC00668 knockout inhibited OSC	VEGF-A	[64]
Osteoclastogenesis	in vitro	Sponging	miR-93-5p	AK131850	cellular proliferation	NA	VEGF-A	[65]
Hepatoblastoma	in vivo	Sponging	miRNA-34a-5p	TUG1	invasion, cellular proliferation	TUG1 knockout inhibited HCC	VEGF-A	[66]
Brain microvascular endothelial cells	in vivo	NA	miR-199a	Snhg1	cellular proliferation		VEGF-A; HIF-1a pathway	[67]
Glioma	in vivo	Sponging	miR-29a	H19	cellular proliferation	knockdown of H19 inhibits glioma	VEGF-A	[68]
Breast cancer	in vivo	NA	miR-3120-5P	WTAPP1	invasion, cellular proliferation	WTAPP1 knockdown and overexpression inhibits breast cancer	PI3K/Akt/mTOR	[69, 70]

MiRNA-299 promotes VEGF-A-dependent angiogenesis in glioblastoma through interacting with lncRNA TUG1. Regression of tumors was induced by therapeutic knockdown of the lncRNA TUG1 [63]. TUG1 can potentially stimulate angiogenesis by interacting with miRNA-34a-5p through a sponging mechanism. Knockdown of the lncRNA TUG1 induces tumor regression [66], and crosstalk regulates VEGF-A to promote invasion and cellular proliferation. Oral squamous cells benefit from increased cell proliferation *via* angiogenesis when LINC00668, another lncRNA, works in concert with miR-297 to negatively regulate the miRNA in a VEGF-A-dependent way. Tumor regression was observed after LINC00668 was knocked down therapeutically [64].

VEGF-A-dependent upregulation of differentiation, migration, and angiogenesis in osteoclastogenesis was also shown in a similar study involving the lncRNA AK131850, which interacts with miR-93-5p via a sponging mechanism. The TGF-β pathway is activated by VEGF-A, which then promotes tumor growth by encouraging angiogenesis [65]. The lncRNA Snhg1 also interacts with miR-199a to trigger VEGF-A-dependent angiogenesis. This feedback loop stimulates the proliferation of microvascular endothelial cells in bone marrow, resulting in angiogenesis [67].

In a VEGF-A-dependent way, lncRNA H19 interacts with miR-29a to promote glioma progression via angiogenesis (cell proliferation and invasion). Regression of gliomas and suppression of angiogenesis can be achieved by knocking down the lncRNA H19 [68]. Similar research identified WTAPP1, a lncRNA that mediates angiogenesis through invasion, cellular differentiation, and proliferation by interacting with miR-3120-5P via a molecular decoy method. Tumor growth in endothelial progenitor cells occurs via the PI3K/Akt/mTOR pathway, and this interaction is reliant on MMP-1 in the autophagy route [69].

To prevent the signaling pathway for tumor progression caused by miRNA and lncRNA crosstalk, MMP − 1 based inhibitors should be utilized. Using immunomodulation of EFGR and TGF-alpha signaling, MMP-1 1-based inhibitor has been used earlier in a breast cancer xenograft model to suppress invasion and metastasis [70]. VASH2 is abundantly expressed in ovarian cell carcinoma, where it stimulates tumor angiogenesis and its knockdown causes tumor regression. In this way, it can serve as a therapeutic molecular target [71].

### Neovascularization and interplay of miRNA and lncRNA

**Fig. 4:**
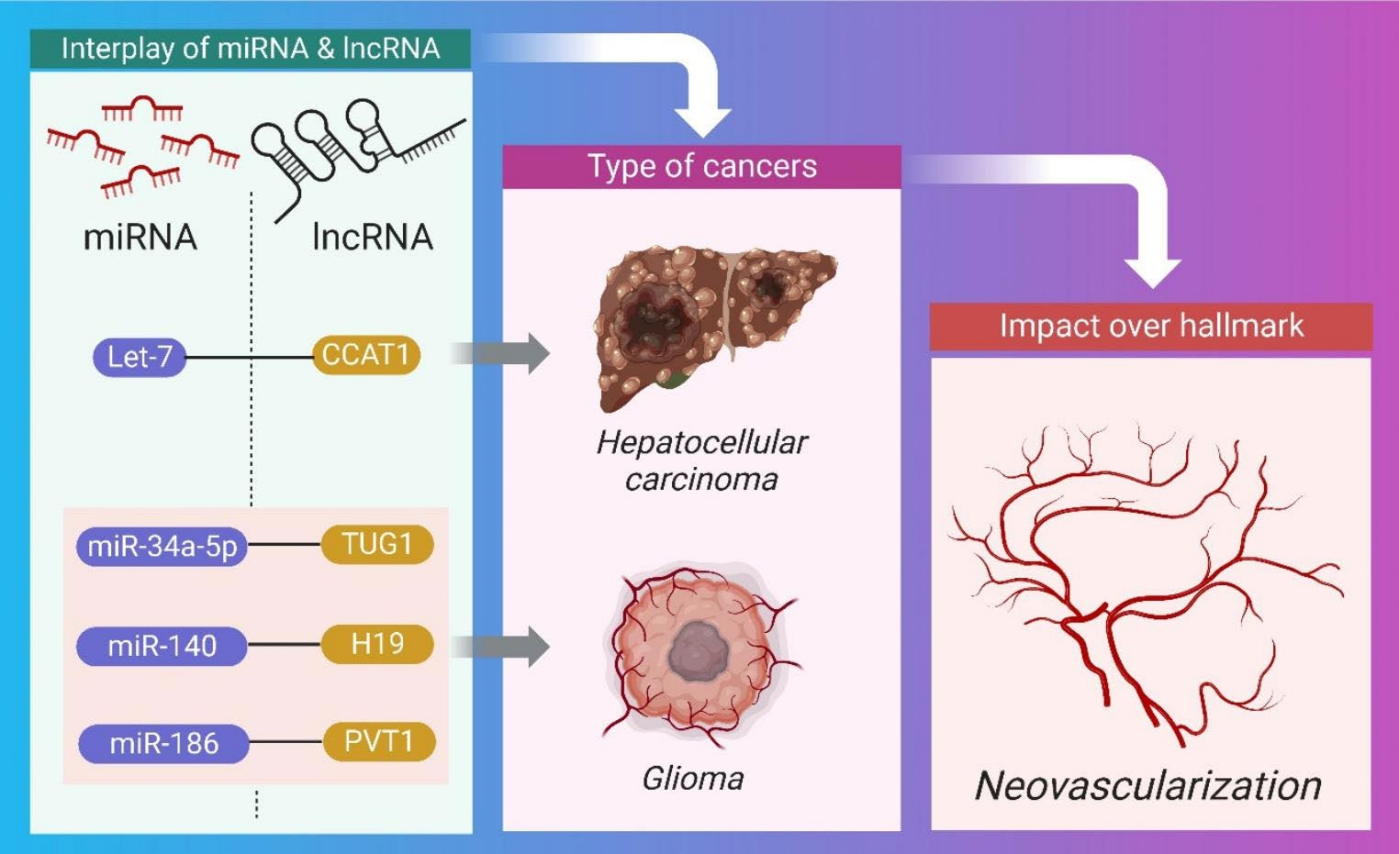
Schematic Representation of the interplay between miRNA and lncRNA causing hepatocellular carcinoma and glioma through neovascularization

Neovascularization is the process of forming new blood vessels from existing ones. Angiogenesis inhibitors and inductors coordinate the procedure after endothelial cell proliferation and migration [72]. In order to receive the nutrients and oxygen they need to grow, tumors need to have close proximity to blood capillaries. Due to oxygen’s long diffusion distance (100-200 m), tumors larger than 12 mm necessitate the development of new blood vessels [73]. In this hypoxic setting, HIF-1 promotes hypervascularization by increasing the production of several growth factors including vascular endothelial growth factor and hepatocyte growth factor [74, 75]. Herein as shown in Fig. 4, and summarized in Table 2, the pre-clinical importance of crosstalk between miRNA and lncRNA impacting neovascularization is being clarified.

**Table 2:** Synopsis of the Crosstalk between miRNA and lncRNA for neovascularization

Cancer type	Type of Pre-clinical	Mechanism	Type of miRNA	Type of lncRNA	Cellular Changes	Therapeutic intervention for tumor regression	Cellular Signaling	Ref.
Hepatocellular carcinoma	In vitro	Sponging	Let-7	CCAT1	Cellular proliferation	knockdown of CCAT1 inhibited HCC	HMGA2, c-Myc	[76]
Glioma	In vitro	NA	miR-34a-5p	TUG1	Cellular proliferation	NA	VEGF-A	[77]
Glioma	In vitro	NA	miR-140	H19	Cellular proliferation	knockdown of H19 inhibited glioma	NA	[78]
Glioma	In vitro	Sponging	miR-186	PVT1	Cellular proliferation, migration	Overexpression of PVTI and miR-186 provides novel targets for glioma anti-angiogenic Therapy	NA	[79]

Through influencing downstream effectors including c-myc and HMGA, which in turn causes neovascularization via cellular proliferation and invasion in hepatocellular carcinoma, the CCAT1 lncRNA promotes tumor growth through a sponging mechanism. Tumors shrank after CCAT1 lncRNA was inhibited [76]. TUG1 is another lncRNA that interacts with miR-34a-5p to promote glioma progression in in vitro and in vivo studies. It’s VEGF-A dependent, therefore it produces neovascularization through invasion and metastasis [77].

The neovascularization of gliomas is facilitated by an interaction between the lncRNA H19 and microRNA-140. Cancer cells shrank after H19 lncRNA was knocked out and p53’s apoptosis-inhibiting protein was targeted therapeutically [78]. Similarly, PVT1, a lncRNA, was found to interact with miR-186 in glioma cells via a negative feedback loop that promotes cell proliferation, motility, and angiogenesis. The upregulation of PVTI and miR-186 may offer new therapeutic targets for anti-angiogenic therapy of gliomas [79].

### EMT and interplay of lncRNA and miRNA

**Fig. 5:**
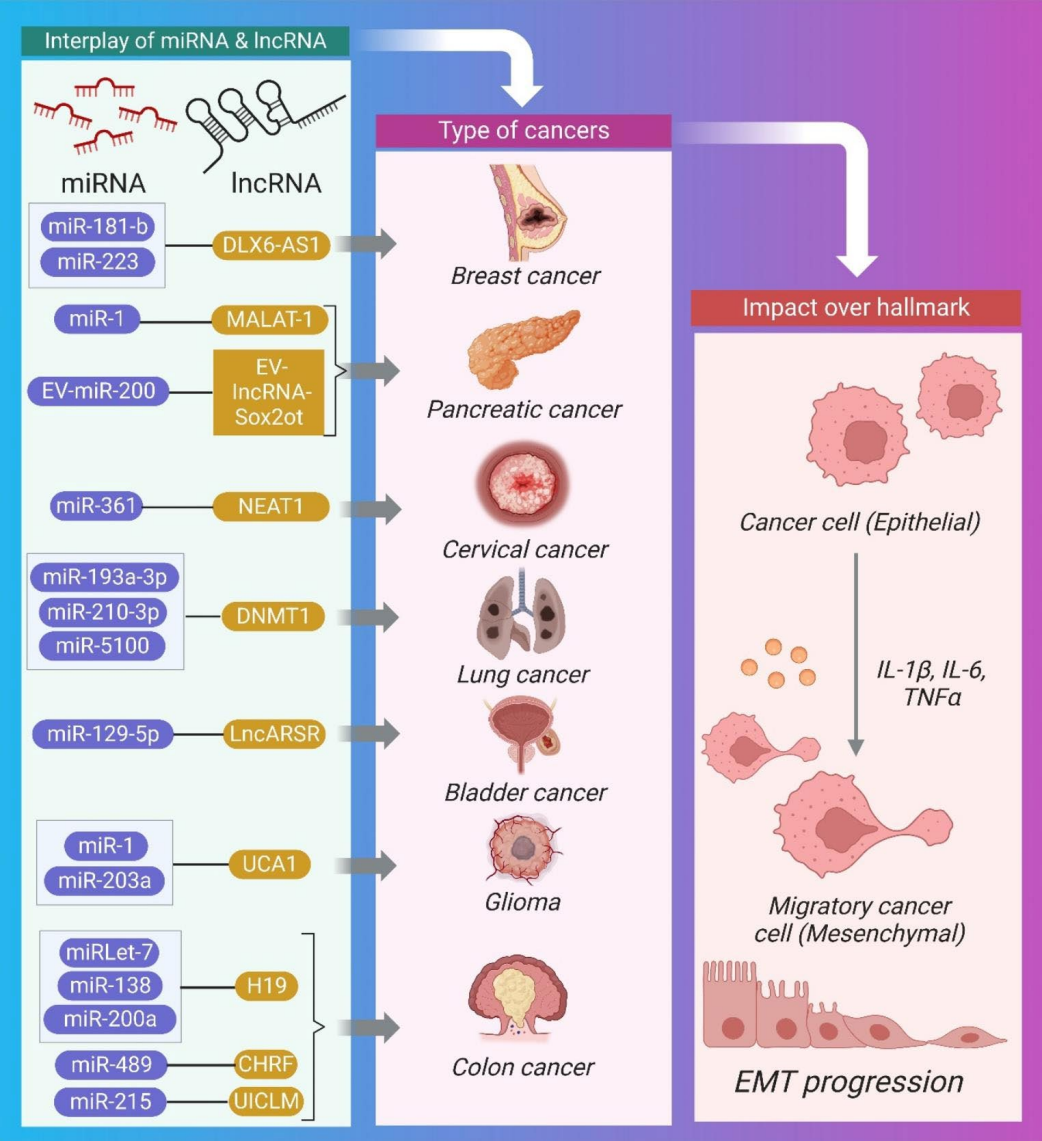
Schematic Representation of the interplay between miRNA and lncRNA causing hepatocellular carcinoma, breast cancer, bladder cancer, pancreatic cancer, lungs cancer cervical cancer, colorectal cancer, and glioma through EMT.

Studies over the past decade have demonstrated the reciprocal role that miRNAs and lncRNAs play in regulating cancer progression via epithelial-mesenchymal plasticity [28, 80, 81]. Because of the interplay between lncRNA and miRNA, EMT is a cancer characteristic that controls the cellular and molecular level processes of malignancies. Whenever lncRNAs interact with miRNAs, their stability is compromised. LncRNAs (ceRNAs) can antagonize microRNAs (miRNAs) by binding to them and preventing them from reaching their targets, as well as binding to mRNAs to compete with miRNAs. In addition, certain lncRNAs produce miRNAs, leading to the silencing of specific mRNAs. Further study of the patterns of interaction between lncRNAs and miRNAs in the biological process of cancer is warranted, as these patterns influence cancer progression [82]. It is essential to regulate the prognosis of early-stage carcinomas by targeting the bidirectional mechanism of epithelial-mesenchymal plasticity [82]. Yet, when cancer cells have spread, inhibiting EMT could be counterproductive because of the positive effects it has on MET [83]. Therefore, it is essential for diagnosis to learn the specific process of secondary site colonization. Here, the impact of miRNA and lncRNA crosstalk on EMT in pre-clinical studies is explained and illustrated (Fig. 5) and summarized (Table 3).

**Table 3:** Synopsis of the Crosstalk between miRNA and lncRNA for EMT

Cancer	Pre-clinical	Mechanism	miRNA	lncRNA	Therapeutic intervention for tumor regression	Cellular Signaling	Ref.
CRC	in vitro	Down regulation	miR-138 and miR-200a	H19	NA	E-cadherin	[84]
Breast cancer	in vivo	Sponging	miR-1	MALAT-1	Knockdown of MALAT1 inhibited breast cancer	NA	[85]
CC	in vitro	Sponging	miR-361	NEAT1		PI3K/AKT	[86]
CRC	in vivo	Sponging	MiR-489	Cardiac hypertrophy-related factor (CHRF)	CHRF knockdown increased the expression of miR-489 and suppressed CRC	TWIST1/EMT	[87]
CRC	in vivo	Sponging	miR-215	UICLM	knockdown of UICLM inhibited CRC	NA	[88]
Lung cancer	in vivo	NA	MiR-193a-3p, miR-210-3p, and miR-5100	DNMT1	NA	STAT3	[89]
Pancreatic ductal adenocarcinoma cells	in vivo	NA	EV-miR-200	EV-lncRNA-Sox2ot	NA	NA	[90]
HCC	in vivo	Sponging	miRlet-7	H19	NA	NA	[91]
Pancreatic cancer	in vivo	Sponging	miR181-b	DLX6-AS1	knockdown of DLX6-AS1 inhibiting PC	E-cadherin upregulated and downregulated MMP2	[92]
Bladder cancer	in vitro	Sponging	miR-129-5p	LncARSR	knockdown of LncARSR inhibited bladder cancer	STAT3; SOX4	[93]
Breast cancer	in vivo	Sponging	miR-223	DLX6-AS1	DLX6-AS1 silencing inhibited breast cancer	Wnt beta -catenin	[94]
Glioma	in vitro	NA	miR-1 and miR-203a	UCA1	knockdown of UCA1 inhibited glioma	downregulation of slug; TGF-beta pathway	[95]

Tumor suppressor miR-217 blocks EMT by preventing EZH2-mediated H3K27me3 through suppression of the lncRNA MALAT-1 via an Ago2-mediated pathway. The therapeutic intervention of downregulating vimentin and N-cadherin and upregulating E-cadherin in HBE cells generated by cigarette smoke extract (CSE) [96] would be consistent with this hypothesis. Moreover, MALAT-1 promotes EMT in triple-negative breast cancer by up-regulating Slug expression via suppression of miR-1 expression via negative feedback loop regulation [85]. This is accomplished by forming complementary base pairs between MALAT-1 and miR-1.

Similarly, lncRNA H19 promotes EMT in CRC by downregulating endogenous target vimentin ZEB1 and ZEB2 through negative regulation of miR-138 and miR-200a [84]. In addition, H19 functions as a sponge interacting with miRlet-7 to promote EMT in HCC [91].

Also, the lncRNA DLX6-AS1 interacts with miR181-b in pancreatic cells, inducing EMT enhancement via cell proliferation, migration, and MMP2 dependence. Knockdown of the lncRNADLX6-AS1 increased E-cadherin and decreased MMP2, preventing malignant cells from undergoing EMT [92]. In addition, EMT in breast cancer is caused by DLX6-AS1 interacting with miR-223 via a sponging mechanism via the Wnt β-catenin signaling pathway in an oncogene HSP90B1-dependent manner [94]. According to the aforementioned research, silencing DLX6-AS1 also causes an increase in apoptosis and a reduction in ESC migration, proliferation, invasion, and EMT.

A related study found that miRNA-lncRNA interactions promoted the development of cancer. Using a sponging mechanism, LncARSR interacts with miR-129-5p, leading to bladder cancer due to SOX4 overexpression during EMT and metastasis. Cancer progression and EMT can be slowed by knocking down this lncRNA, making it a potential therapeutic target [93]. UCA1 is another lncRNA that induces glioma growth via EMT via TGF-β and functions as a competitive RNA for miR-1 and miR-203a. Tumor regression can be induced by knocking down UCA1, which leads to the downregulation of slug, a molecular effector protein [95]. The aberrant production of IL-6 promotes EMT in HCC via activating the STAT3 pathway through lncTCF7 [97]. IL-6 in the tumor microenvironment promotes lncTCF7 by activating STAT3.

MicroRNAs play a role in lncRNA-dependent gene regulation during EMT in CC metastases [98]. NEAT1 is a lncRNA that mediates EMT through invasion in CC [86]. It does this by interacting with the miR-361 and adversely impacting it in an HSP90 signaling-dependent manner. In addition, the lncRNA cardiac hypertrophy-related factor (CHRF) negatively influences the anti-metastatic factor miR-489, hence promoting metastasis and EMT [87]. The lncRNA UICLM interacted with miR-215, which promotes cellular invasion, migration, and proliferation through EMT in a ZEB2-dependent fashion [88].In hypoxic bone marrow mesenchymal stem cells (BMSCs), the EV-miRNAs miR-5100, miR-210-3p, and MiR-193a-3p interact with the lncRNA DNMT1 to promote EMT, migration, and invasion in lung cancer cells. [89]. The dissemination of malignant phenotypes among diverse populations of cancer stem cells is facilitated by the exchange of EV-RNAs within tumors. By transferring the metastatic capacity to nearby or distant weakly metastatic BC cells, EV-miR-200 from highly metastatic BC cells promotes EMT and lung colonization [99, 100]. MiR-200c is targeted, Sox2 is increased, and EV-lncRNA-Sox2ot is released from highly invasive pancreatic ductal adenocarcinoma cells, inducing EMT and stemness in less invasive recipient cells, and ultimately causing in vivo tumor metastasis [90]. To increase pancreatic cancer cell motility, invasion, EMT, and lung metastasis by secreting IL-10, TGF-β, and arginase-1, EV-miR-301a interacts with HOTAIR and MIR31HG from hypoxic pancreatic cancer cells and causes macrophage M2 polarization [101].

### Vascular mimicry and interplay of miRNA-lncRNA

Similar to the embryonic vascular network, tumor tissue can receive plasma and red blood cells via microcirculatory channels made of extracellular matrix in a process known as vascular mimicry (VM) [102][102–104, 109]. VM is unique because it resembles embryonic vasculogenesis processes, which suggests that tumor cells can be reverted to an undifferentiated, embryonic-like phenotype to supply nutrients for tumor growth in hypoxic environments in lung, colorectal, bladder, osteosarcoma, prostate, breast, ovarian, and melanoma cancers, and to facilitate invasion and metastasis in these diseases. VM causes high levels of matrix metalloproteinase-9 (MMP-9), metalloproteinase-2 (MMP-2), vimentin (VIM), fms-like activating kinase (FAK), ephrin-A2 (EphA2), vascular endothelial growth factor (VEGF), transforming growth factor beta 1 (TGF-ß1), Dickkopf-1 (DKK-1), maspin (maspin), lamin. Here, we are clarifying the pre-clinical significance of crosstalk between miRNA and lncRNA as it relates to neovascularization and summarizing this interaction in Table 4.

**Table 4:** Synopsis of the Crosstalk between miRNA and lncRNA for Vascular mimicry

Cancer	Pre-clinical	Mechanism	miRNA	lncRNA	Cellular Changes	Therapeutic intervention for tumor regression	Cellular Signaling	Ref.
Glioma	in vivo	Sponging	miR-373	HOXA-AS2	Cellular proliferation	knockdown of HOXA-AS2 inhibited glioma	PIP3 and EGFR	[103]
Glioma, Breast cancer	in vivo	Negative regulation	miR-539-5p	TWIST1	Migration, invasion	knockdown of TWIST1 inhibited glioma	MMP-2 and MMP-14	[104, 105]
Hepatocellular carcinoma	in vitro	Sponging	miR-31-3p, miR-30e-5p, miR-519c-5p, miR-520c-5p, miR-29b-1-5p, and miR-92a-1-5p	n339260	Stemlike features and VM development; Cellular proliferation	Knockdown of LINC00339 inhibited glioma cell	MMP-2, MMP-9, and MMP-14	[106]
Lung cancer	in vitro	Sponging	miR-145-5p	MALAT1	VM causing metastasis	knockdown of molecular effector molecule Erβ inhibited lungs cancer	NEDD9	[107]

Neither microRNAs nor long noncoding RNAs have been overlooked in their roles as master controllers of cancer progression in VM. In a murine model of glioblastoma, HOXA-AS2 is a lncRNA that acts as a sponge for miR-373 through MMP-9, MMP-2, or VE-cadherin in a PIP3-kinase, serine/threonine kinase, and EGFR EGFR-dependent way. Therapeutically, HOXA-AS2 knockdown can be used to reduce glioma progression [103]. Similarly, MALAT1 interacted with miR-145-5p in lung cancer patients, inducing metastasis in an aEr dependent manner, via sponging off via NEDD9 signaling for VM. Therapeutic intervention against lung cancer can be achieved through the knockdown of molecular effector molecules like Er [107].

Glioma cells migrate and invade when the lncRNA TWIST1, upon LINC00339 polymorphism, interacts with miR-539-5p, negatively impacting the miRNA for VM in an MMP-2 and MMP-14 dependent way. Therapeutically, TWIST1 knockdown can lead to glioma regression [104]. In addition, in an in-vitro investigation against breast cancer cells, TWIST1 interacted with miR-430-3p, negatively affecting the miRNA in a TP73-AS1-dependent mechanism for VM. Therapeutically, breast cancer regresses when the lncRNA TWIST1 is knocked down [105].

Similar research found that the lncRNA n339260 correlated with the expression of the pluripotency-maintaining molecules c-myc, SOX2, and Nanog, and interacted with multiple miRNAs (miR-92a-1-5p, miR-29b-1-5p, miR-520c-5p, miR-519c-5p, miR-30e-5p, and miR-31-3p) for stem-like features and VM development.

### Impact of crosstalk of miRNA and lncRNA on inhibition of apoptosis

**Fig. 6:**
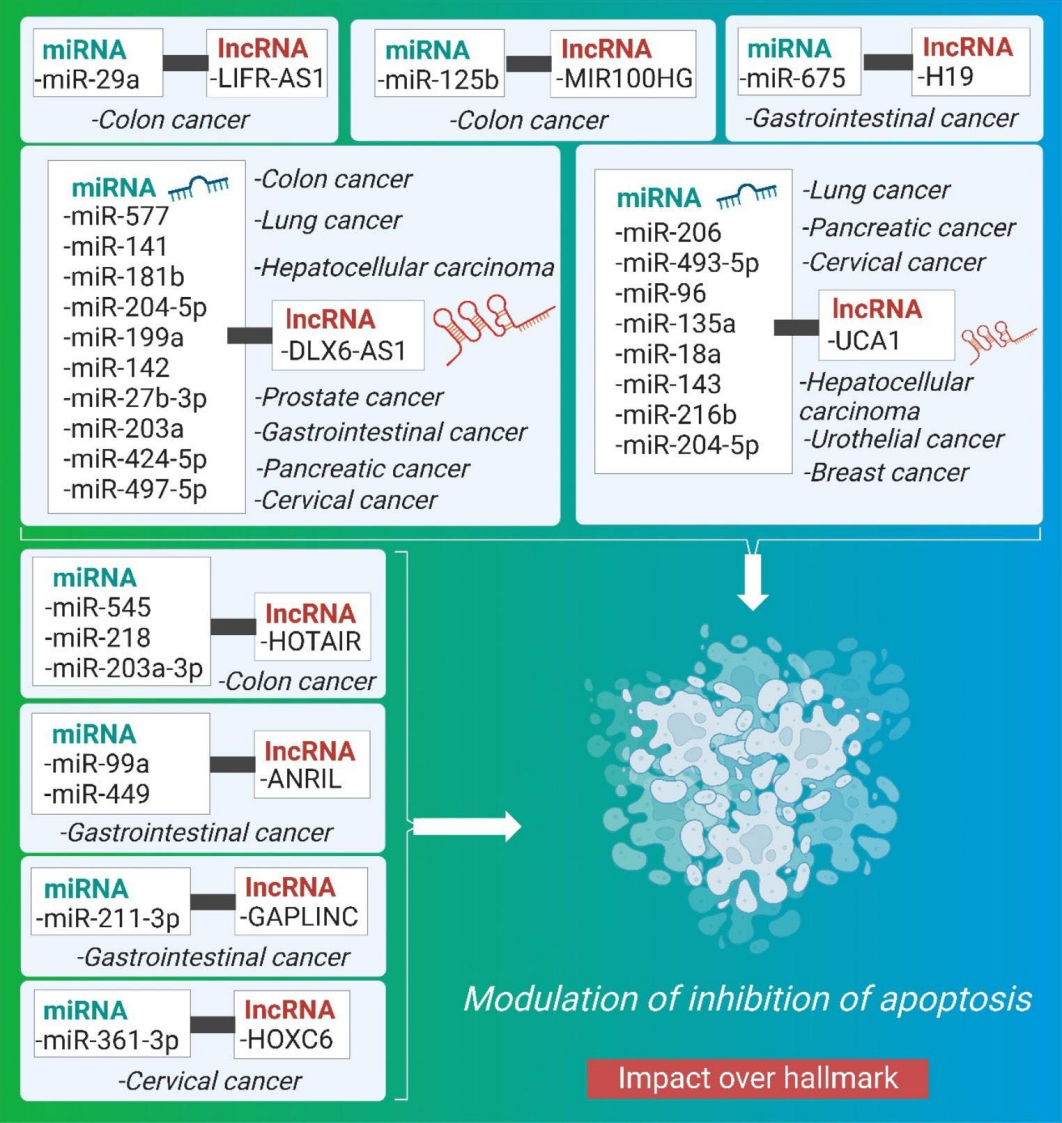
Schematic representation of the interplay between miRNA and lncRNA causing cancer progression through modulation of inhibition of apoptosis

Inhibition of apoptosis is a well-coordinated cellular hallmark that occurs in a variety of physiological circumstances in cancer cells to evade the apoptotic response [108, 109]. Gene inactivation at the p53 locus results in epigenetic regulation that increases the expression of anti-apoptotic proteins and decreases the expression of pro-apoptotic proteins, making the cell resistant to a wide range of apoptotic triggers. Recent research has demonstrated that ncRNAs have a critical regulatory role in the prevention of apoptosis. Several of these RNAs’ key roles in oncogenic processes are in controlling cell apoptosis. [110]. Overexpression of lncRNA can reduce the expression of necroptosis-related proteins and block the extrinsic apoptosis pathway by downregulating membrane receptors. [111]. By modulating the expression of effector molecules involved in apoptosis signaling pathways, lncRNA and miRNA inhibits apoptosis through interacting among themselves. Herein, the pre-clinical significance of crosstalk between miRNA and lncRNA affecting inhibition of apoptosis is being elucidated, illustrated in Fig. 6, and are summarized in Table 5.

**Table 5:** Synopsis of the Crosstalk between miRNA and lncRNA for influencing inhibition of apoptosis

Cancer type	Type of Pre-clinical	Mechanism	miRNA	lncRNA	Therapeutic intervention for tumor regression	Cellular Signaling /Molecular effectors	Ref.
CRC	in vitro	Sponging	miR-577	DLX6-AS1	inhibition of PI3K/mTOR pathway; overexpression of miR-577 inhibited CRC	p-P13K and p-AKT	[112]
Urothelial carcinoma	in vitro	Negative regulation	miR-204e5p	UCA1	UCA1 knockdown and miR-204-5p overexpression induced downregulation of endogenous expression of miR-204-5p target genes in HCT116 cells.	UCA1/miR-204-5p ceRNA	[113]
CRC	in vitro	NA	miR-203a-3p, miR-545, and MiR-218	HOTAIR	Both HOTAIR knockdown and miR-203a-3p overexpression in CRC cell lines led to inhibited CRC	Wnt- b-catenin, EGFR	[114–116]
CRC	in vitro	Sponging	miR-141	DLX6-AS1	HOTAIR knockdown dramatically inhibited cell viability and induced G1-phase arrest by promoting miR-218 expression.	NF-κB/TS	[117]
CRC	in vitro	Sponging	miR-369e3p	OIP5-AS1	NA	NA	[118]
CRC	in vitro	Sponging	miR-29a	LIFRAS1	LIFR-AS1 knocking down remarkably promoted the cell proliferation of HCT116 cells whereas miR-29a significantly inhibited HCT116 cell proliferation	Wnt/β-catenin	[119] [120]
CRC	in vitro	Sponging	miR-125b	MIR100HG	NA	Wnt/β-catenin	[47]
Breast Cancer	in vitro	NA	miR-18a	UCA1	knockdown of UCA1 inhibited breast cancer	NA	[121]
Lung cancer	in vitro	Sponging	miR-142 and miR-27b-3p	DLX6-AS1	knockdown of lncRNA and up-regulation of miR-142	NA	[122, 123]
Lung Cancer	in vitro	NA	miR-193a	UCA1	knocking down miR-193a-3p had the opposite effect on cell proliferation	PTEN/PI3K	[124]
Bladder and lung cancers	in vitro	NA	miR-144	UCA1	Knockdown of UCA1 significantly inhibited lung cancer	NA	[125, 126]
Lung Cancer	in vitro	NA	miR-143a	UCA1	NA	NA	[127]
Hepatocellular carcinoma	in vitro	Sponging	miR-203a and miR-424-5p	DLX6-AS1	Knockdown DLX6-AS1 suppressed HCC	EGFR/PI3K/AKT	[128, 129]
Hepatocellular carcinoma	in vitro	Sponging	miR-216b	UCA1	NA	MAPK, ERK	[130, 131]
Pancreatic cancer	in vitro	NA	miR-181b	DLX6-AS1	Knockdown of DLX6-AS1 inhibited PC	NA	[132]
Prostate cancer	in vitro	Sponging	miR-497-5p	DLX6-AS1	DLX6-AS1 significantly reduced PC	DLX6-AS1/miR-497-5p/FZD4/FZD6/Wnt/β-catenin	[133]
Pancreatic cancer	in vitro	Sponging	miR-96 and miR-135a	UCA1	NA	NA	[134]
Gastric cancer	in vitro	NA	miR-675	H19	H19 knockdown inhibited GC	NA	[135, 136]
Gastric cancer	in vitro	NA	miR-211-3p	GAPLINC	Knockdown of GAPLINC inhibited GC	NA	[137, 138]
Gastric cancer	in vitro	NA	miR-99a and miR-449	ANRIL	knockdown of ANRIL inhibited GC	NA	[139]
CC	in vitro	Sponging	MiR-199a	lncRNA DLX6-As1	Knockdown of DLX6AS1 inhibited CC	NA	[140]
CC	in vitro	NA	miRNA206 and miR-493-5p	UCA1	Knockdown of UCA1 inhibits CC	VEGF	[141, 142]
CC	in vitro	Sponging	miR-361-3p	HOXC6	knockdown of BBOX1-AS1 inhibited CC	NA	[143]
CC	in vitro	Sponging	miR-143-3p	OIP5-AS1	NA	OIP5-AS1/miR-143-3p-ROCK1	[144]

The lncRNA DLX6-AS1 negatively regulated miR-577, which in turn promoted CRC progression by inhibiting apoptosis and metastasis and elevating p-P13K and p-AKT signaling pathway levels. Tumors shrank after a surge in miR-577 expression was triggered by blocking the PI3K/mTOR signaling pathway [112]. In addition, DLX6-AS1 interacts with miR-142 in a PRR11 gene-dependent manner, suppressing apoptosis and metastasis in the process of developing lung cancer. Tumor regression and miR-142 upregulation both resulted after lncRNA inhibition knock-down. By promoting cell proliferation and inhibiting apoptosis [122, 123], miR-27b-3p contributes to the development of lung cancer. [122, 123].

In addition, DLX6-AS1 induced hepatocellular carcinoma via interacting with miR-203a, which in turn inhibited apoptosis, cellular proliferation, and metastasis in an MMP MMP-dependent manner [128, 129]. Similarly, lncRNA DLX6-AS1 induces hepatocellular cancer by suppressing apoptosis through the STAT pathway after binding with miR-424-5p in a WEE1-dependent manner. Hence, DLX6-AS1 knockdown represents a potential therapeutic strategy for the treatment of HCC. In a fibroblast growth factor Receptor 1–dependent manner, the lncRNA UCA1, when interacting with miR-216b, promotes suppression of G0/G1 cell cycle arrest, hence inhibiting apoptosis and metastasis in hepatocellular carcinoma cells [130, 131]. A similar study found that the zinc finger E-box-binding protein DLX6-AS1, upon interacting with miR-181b, promoted cell proliferation, migration, metastasis, and inhibition of apoptosis in pancreatic cancer via up-regulation of the Wnt/beta-catenin signaling pathway and the FZD4/FZD6 signaling pathway (ZEB2). Tumor regression was observed after DLX6-AS1 was knocked down, suggesting that this process could be used therapeutically. Interaction between DLX6-AS1 and miR-181b facilitated cell proliferation, migration, metastasis, and apoptosis suppression [132]. The MAP4K1 protein inhibits apoptosis and metastasis in gastric cancer, and this has been linked to its upregulation of the interaction of lncRNA DLX6-AS1 with miRNA expression [145–147]. Via a direct interaction with miR-204-5p, DLX6-AS1 suppressed EMT and invasion in GC cells by blocking apoptosis and metastasis in OCT1-dependent fashion [148]. MiR-199a inhibits apoptosis and metastasis in CC through an interaction with lncRNA DLX6-As1 [140]. This effect is FUS-dependent.

Another lncRNA, urothelial carcinoma-associated 1 (UCA1), has been shown to interact with miR-204e5p via negative regulation to up-regulate CREB1 expression, which in turn can promote cell proliferation and treatment resistance to 5-fluorouracil via prevention of apoptosis and metastasis [113]. Inhibition of apoptosis and metastasis in CRC can also be attributed to the lncRNA HOTAIR [114–116], which interacts with several miRNAs including miR-218, miR-545, and miR-203a-3p via Wnt- β-catenin, EGFR signaling. More so, UCA1 regulates the cell cycle by blocking apoptosis when it interacts with miR-18a. Inhibiting the expression of the lncRNA UCA1 may prove useful in the therapeutic management of breast cancer [121]. In addition, UCA1 interacts with miR-193a, leading to lung cancer via cell proliferation, apoptosis suppression, and metastasis in an ERBB4 and HMGB1-dependent way [124]. The homeobox 3 gene promotes invasion, suppression of apoptosis, and metastasis in bladder and lung malignancies [125, 126], and UCA1 interacts with miR-144 via genes involved in pre-b cell leukemia. Similarly, UCA1 inhibits apoptosis in lung cancer by causing a G2/M cell cycle stop through a negative-influencing interaction with miR-143 [127]. Similar research has found that UCA1 promotes cervical cancer glycolysis by inhibiting apoptosis via a sponging mechanism with miR-493-5p [142]. CC is brought on by UCA1’s negative feedback mechanism with miRNA-206, which promotes cell development and inhibits apoptosis in a vascular endothelial growth factor (VEGF)-dependent manner [141]. Through interacting with miR-96, the lncRNA UCA1 promoted cellular proliferation and suppressed apoptosis and metastasis in pancreatic cancer. UCA1 promotes cell proliferation and suppresses apoptosis by interacting with miR-135a in pancreatic cancer [134].

Another lncRNA (Opa-interacting macromolecule five antisense polymer one; OIP5-AS1) interacts with microRNA-369e3p to suppress apoptosis and metastasis in colorectal cancer (CRC) cells and increase expression of dual-specificity tyrosine-regulated enzyme 1 A (Dyrk1A) RNA. OIP5-AS1 also interacted with miR-143-3p via a molecular sponge method, preventing apoptosis, cell proliferation, and metastasis in a ROCK1-dependent way [144].

Overexpression of programmed cell death 4 (PDCD4) is caused by the lncRNA MEG3, which interacts with miR-141 through a competitive binding mechanism, making CRC cells resistant to oxaliplatin [117]. Furthermore, lncRNA LIFR antisense polymer one (LIFRAS1) induced drug resistance in CRC by interacting with miR-29a in TNFAIP3 [120]. In addition, HOTAIR is another another lncRNA that, after interacting with miRNA, increases GC advancement via invasion and prevention of apoptosis. Therapeutic applications for lncRNA silencing have been proposed [149]. H19, a lncRNA, interacts with miR-675 to cause GC by blocking apoptosis and metastasis in a Runt Domain Transcription Factor 1 (RUNX1) and Calneuron 1 dependent manner [135, 136].

GAPLINC, another lncRNA, interacts with miR-211-3p in a CD44 CD44-dependent way, inducing GC by inhibiting apoptosis and metastasis via a molecular decoy mechanism [137, 138]. For this reason, knockdown of GAPLINC lncRNA could be used as a therapy for GC [150]. Similar research has shown that the lncRNA ANRIL interacts with the miRNAs miR-99a and miR-449 to prevent apoptosis and metastasis, hence promoting the development of GC. Tumors regressed once NARIL was knocked down, suggesting that this strategy could be used in therapy [139]. Through suppressing apoptosis and metastasis, the lncRNA HOXC6 interacts with the microRNA miR-361-3p to promote CC progression [143].

### Impact of crosstalk of miRNA and lncRNA on metastasis

**Fig. 7:**
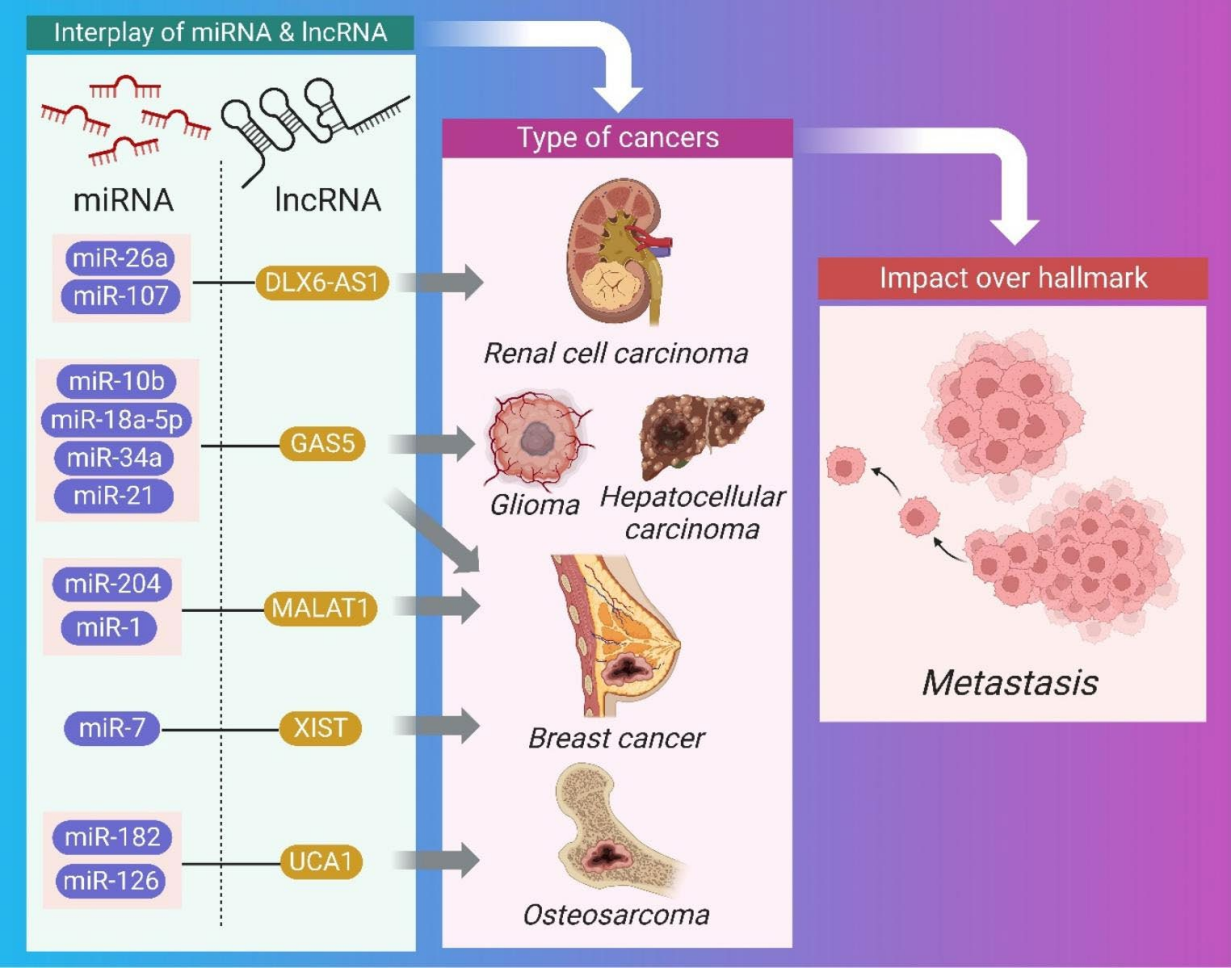
Impact of crosstalk between miRNA and lncRNA on various cancers affecting metastasis

Malignant malignancies are characterized by metastasis, the spread of tumors to distant tissues [151]. Cancer cells leave their main location to circulate in the circulation, adapting to new biological circumstances in a secondary site in order to withstand pressure in blood arteries and dodge immuno-stimulation by immune cells [152, 153]. Table 6 summarizes the research on how miRNAs and lncRNAs interact with one another to regulate protein-coding genes involved in cancer metastasis [154, 155]. In this article, we are elucidating the pre-clinical relevance of crosstalk between miRNA and lncRNA impacting prevention of apoptosis (Fig. 7).

**Table 6:** Synopsis of the Crosstalk between miRNA and lncRNA for influencing metastasis

Cancer	Pre-clinical	Mechanism	miRNA	lncRNA	Therapeutic intervention for tumor regression	Cellular Signaling	Ref.
Renal cell carcinoma	in vivo	Down regulation	miR-26a	DLX6-AS1	knockdown of DLX6-AS1 inhibited RCC	PTEN	[156]
Brain cancer and glioma	in vivo	Down regulation	miR-107	DLX6-AS1	Knockdown of miR-107 inhibited brain cancer	BDNF-dependent	[157]
Brain cancer and glioma	in vivo		miR-10b	GAS5	NA	PIP3K, AKT, ERK and MEK	[158]
Glioma	in vivo	Sponging	miR-18a-5p	GAS5	overexpression of GAS5 inhibits glioma cells	PTEN–PI3K–AKT– mTOR	[159]
Renal cell carcinoma and hepatocellular cell carcinoma	in vivo	Sponging	MiR-34a	GAS5	NA	p53	[160]
Breast cancer	in vivo	NA	miR-21	GAS5	Knockdown of miR-21 inhibited breast cancer.	HER2*/*neu	[161]
Osteosarcoma	in vivo	NA	miR-182	UCA1	Knockdown of UCA1 inhibited OS.	PTEN/AKT	[160, 162]
Leukemia	in vivo	Sponging	miR-126	UCA1	Knockdown of UCA1 inhibited ML cells in vitro.	PI3K/AKT and JAK/STAT	[163]
Breast cancer	in vivo	Sponging	miR-204 and miR-1	MALAT1	knockdown of MALAT1 inhibited breast cancer	MAPK-ERK	[85, 164]
Breast cancer	NA	NA	miR-7	X-inactive-specific transcript (XIST)	NA	NA	[165]

Renal cell cancer was triggered by DLX6-AS1 through the PTEN pathway and metastasis as a result of its interaction with miR-26a via a negatively impacting mechanism. Tumors may be regressed by silencing the lncRNA DLX6-AS1 [156]. In addition developing brain cancer and glioma through metastasis, DLX6-AS1 interacts with miR-107 in a BDNF-dependent manner, negatively impacting processes.

Brain cancer and glioma are the result of another miRNA-lncRNA interaction: GAS5, which interacts with miR-10b and promotes metastasis by activating mitogen-activated protein kinase kinases (MEK), protein kinase B (AKT), phosphoinositide 3-kinase (PI3K), and extracellular signal-regulated kinase (ERK), and PTEN pathway. Tumor regression may be induced by therapeutic suppression of phosphorylation of PIP3K, AKT, ERK, and MEK pathways [158]. Moreover, GAS5 interacts with miR-18a-5p to promote glioma growth via a mimic mechanism that ultimately leads to metastasis [159]. In addition, GAS5 interacts with MiR-34a in a manner that promotes the spread of renal cell carcinoma and hepatocellular cell carcinoma [160].

Prostate cancer metastasis and cellular proliferation were caused by the lncRNA UCA1 interacting with miRNA [166]. This was dependent on the transcription factors Sirt1 and ATF2. Also, UCA1 interacts with miR-182, leading to osteosarcoma via cellular proliferation and metastasis in a TIMP2-dependent manner [160, 162]. In a Ras-related toxin substrate 1–dependent way, UCA1 interacts with miR-126 via an endogenous sponge mechanism in leukemia [163].

In a Slug and ZEB2 protein-dependent way, the lncRNA MALAT1 interacts with miR-204, leading to breast cancer growth via metastasis. MALAT1 knockdown led to Slug inhibition, which in turn led to tumor regression via increased apoptosis and metastasis [164]. In addition, MALAT1 interacts with miR-1 to promote the spread of breast cancer, a process that is slug-dependent [85].

### Impact of crosstalk of miRNA and lncRNA on invasion

**Fig. 8:**
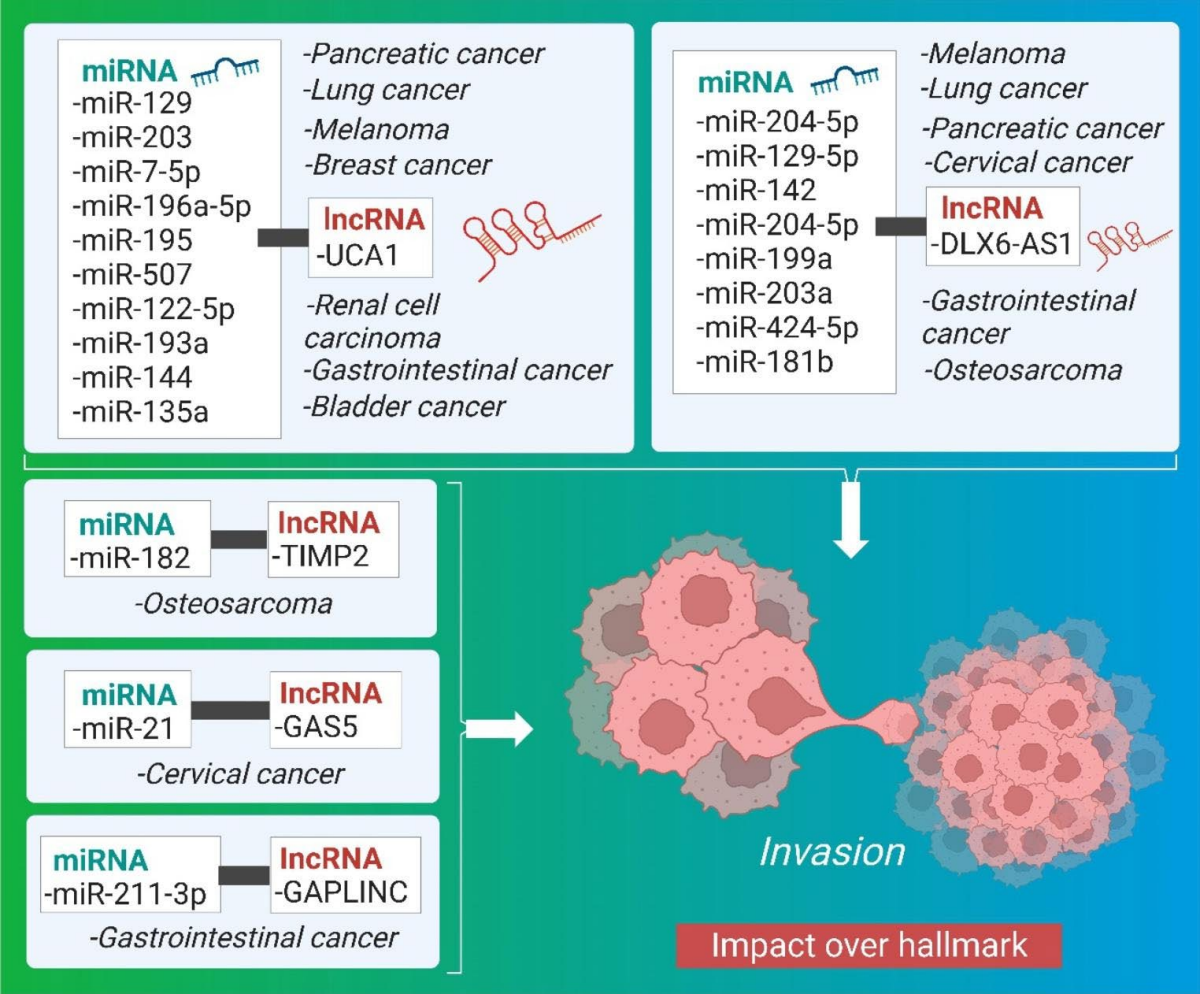
Impact of crosstalk between miRNA and lncRNA on various cancers affecting invasion

Physical, biological, and molecular variables adapt with cancer progression via signaling pathways to enhance cytoskeletal dynamics for cell-matrix and cell-cell junction turnover, resulting in cell migration into adjacent tissue. [167, 168]. By decreasing or removing intercellular adhesion molecules, the separation of malignant cells from the tumor bulk increases invasiveness. They induce abnormally high motility by allowing the cells to penetrate the structural features of the surrounding stroma [169]. Table 7 summarizes how the interaction between miRNA and lncRNA plays a crucial role in cancer invasion, while the pre-clinical significance of crosstalk between miRNA and lncRNA affecting inhibition of apoptosis, illustrated in Fig. 8.

**Table 7:** Synopsis of the Crosstalk between miRNA and lncRNA influencing invasion

Cancer	Pre-clinical	Mechanism	miRNA	lncRNA	Therapeutic intervention for tumor regression	Cellular Signaling	Ref.
Renal cell carcinoma	in vitro	Sponging	miR-129	UCA1	CA1 knockdown in RCC cells	NA	[170]
Gastrointestinal cancer	in vitro	Sponging	miR-203 and miR-7-5p	UCA1	Knockdown of UCA1 inhibited GIC	AKT/GSK-3β	[171, 172]
Bladder Cancer	in vitro	Sponging	miR-196a-5p	UCA1	Knockdown of UCA1	NA	[173, 174]
Bladder cancer	in vitro	NA	miR-195	UCA1	knockdown of UCA1 inhibited bladder cancer	MiR-195/ARL2	[175–177]
Melanoma	in vitro	NA	anti-miR-185-5p	UCA1	NA	NA	[178, 179]
Melanoma	in vitro	NA	FOXM1 (miR-507)	UCA1	NA	NA	[180, 181]
Osteosarcoma	in vitro	NA	miR-182	TIMP2	NA	NA	[162]
Breast cancer	in vitro	Sponging	miR-122-5p	UCA1	NA	FGFR1 and ERK	[182]
Osteosarcoma	in vitro	NA	miR-129-5p	DLX6-AS1 and DLK1	DLX6-AS1 knockdown decreased tumor sphere number and size in MG63 and U2OS cells	Wnt-beta pathway	[183]
Urothelial carcinoma	in vitro	Negative Regulation	miR-204e5p	UCA1	UCA1 knockdown and miR-204-5p overexpression induced downregulation target genes in HCT116 cells.	UCA1/miR-204-5p ceRNA	[113]
CRC	in vitro		miR-203a-3p, miR-545 and miR-218	HOTAIR	Both HOTAIR knockdown and miR-203a-3p overexpression inhibited CRC	Wnt- b-catenin, EGFR	[114, 115]
Colon cancer, Gastric adenocarcinoma	in vitro	NA	miR-34a	lnc34a	NA	PI3K/Akt/mTOR	[36, 184, 185]
CRC	in vitro	Sponging	miR-369e3p	OIP5-AS1	NA	NA	[119]
CRC	in vitro	Sponging	miR-29a	LIFRAS1	miR-29a significantly inhibited CRC	Wnt/β-catenin	[120]
Lung cancer	in vitro	Sponging	miR-142	DLX6-AS1	knockdown of lncRNA and up-regulation of miR-142inhibited LC	NA	[122, 123]
Lung cancer	in vitro		miR-193a	UCA1	knocking down miR-193a-3p	PTEN/PI3K	[124]
Bladder and lung cancers	in vitro	NA	miR-144	UCA1	Knockdown of lncRNA UCA1 inhibited lung cancer	NA	[125, 126]
Lung cancer	in vitro	NA	miR-143	UCA1	NA	NA	[127]
Hepatocellular carcinoma	in vitro	Sponging	miR-203a and miR-424-5p	DLX6-AS1	Knockdown DLX6-AS1 inhibited HCC	EGFR/PI3K/AKT	[128, 129]
Pancreatic cancer	in vitro	NA	miR-181b	DLX6-AS1	NA	NA	[132]
Pancreatic cancer	in vitro	Sponging	miR-497-1	DLX6-AS1	knockdown of DLX6-AS1 inhibited PC	FZD4/FZD6/Wnt/β-catenin	[133]
Pancreatic cancer	in vitro	Sponging	miR-96 and miR-135a	UCA1	knockdown of UCA1 inhibited PC	NA	[134, 186]
Gastric cancer	in vitro	NA	miR-204-5p	DLX6-AS1	Knockdown of DLX6-AS1 inhibited GC.	GSK3-β catenin; upregulated E-cadherin protein; downregulated N-cadherin and vimentin protein	[145–147]
Gastric cancer	in vitro	Sponging	miR-331-3p	HOTAIR	knockdown of HOTAIR inhibited GC	NA	[187]
Gastric cancer	in vitro		miR-675	H19	H19 knockdown inhibited GC	NA	[135, 136]
Gastric cancer	in vitro	NA	miR-211-3p	GAPLINC	Knockdown of GAPLINC	NA	[137, 138, 150]
CC	in vitro	Sponging	miR-199a	DLX6-As1	Knockdown of DLX6AS1 inhibited CC	NA	[188]
CC	in vitro	Sponging	miR-143-3p	OIP5-AS1	NA	OIP5-AS1/miR-143-3p-ROCK1	[144]
CC	in vitro	Sponging	miR-21	GAS5	knockdown of GAS5 inhibited CC	NA	[189]

LncRNA UCA1 interacts with miR-129 via RCC invasion via the SOX4 pathway. The lncRNA UCA1 knockdown caused cancer regression and might potentially be employed for therapeutic interventions [170]. Furthermore, in gastrointestinal cancer progression, UCA1 promotes invasion, cell migration, and apoptosis by interacting with the two miR-203 and miR-7-5p in a zinc finger and homeobox2 homeobox2-dependent way via EGFR [171, 172]. Additionally, UCA1 interacts with miR-196a-5p for bladder cancer through boosting cancer cell invasion by targeting Fascin homologs [173, 174]. UCA1 aided in the regulation of glutamine metabolism and the production of reactive oxygen species (ROS) in bladder cancer cells [175, 176]. Similarly, UCA1 interacted with miR-195 to promote mitochondrial function in ADP-ribosylation factor-like protein 2 dependent ways [177]. UCA1 also interacted with an anti-miR-185-5p gene to increase melanoma cells proliferation, migration, and invasion via control of the Wnt-β-catenin signaling pathway [178, 179]. FOXM1, also known as miR-507, is a tumor suppressor that interacts with the lncRNA UCA1, promoting accelerated melanoma development by targeting the G2/M phase [180, 181]. UCA1 also interacted with miR-122-5p to promote breast cancer growth via invasion and cellular proliferation by up-regulating its target genes via RNA binding protein and inhibiting the HER3 kinase UTR [182]. LncRNA UCA1 inhibits miR-204e5p expression by a similar mechanism that can up-regulate CREB1 expression, resulting in cell proliferation and 5-fluorouracil resistance [113]. UCA1 also aided pancreatic cancer growth by decreasing miR-135a expression [134].

MIIP binds to histone deacetylase 6 (HDAC6) and inhibits cell motility by lowering protein stability, resulting in a positive feedback loop between FOXO1 and GAS5 [190]. TIMP2 is a lncRNA that interacts with miR-182 to drive osteosarcoma invasion in an MMP MMP-dependent way [162].

DLX6-AS1 is another lncRNA that, upon association with miR-129-5p, functions in the formation of OS via the Wnt-beta signaling pathway by overexpression of Delta Like Non-Canonical N-Lept 1. (DLK1). By decreasing the Wnt-beta signaling pathway, DLX6-AS1 and DLK1 hindered self-renewal and stability [183]. Furthermore, DLX6-AS1 increases invasion in lung malignancies via negative regulation via crosstalk with miR-142. The lncRNA knockdown produced tumor regression and can thus be employed therapeutically. The lncRNA DLX6-AS1 is up-regulated in human liver cells and tissues after interacting with miR-203a, promoting invasion in human hepatocellular carcinoma in an MMP2 MMP2-dependent manner. By knocking off the lncRNADLX6-AS1, it suppressed cell proliferation and invasion [128, 129].

Moreover, DLX6-AS1 interacts with miR-424-5p via the STAT3 signaling pathway, promoting invasion in WEE1-dependent hepatocellular cancer [191]. When DLX6-AS1 interacted with miR-181b, it promoted pancreatic invasion via an up-regulated Wnt-β-catenin FZD4/FZD6 signaling pathway that was dependent on the zinc finger E-box-binding protein (ZEB2). The suppression of lncRNA reduced the levels of ZEB2, a regulatory protein involved in the induction of invasion in pancreatic cancer [132]. DLX6-AS1 also interacts with miR-497-1 via an endogenous sponge mechanism in a FZD4 dependent way, inducing pancreatic cancer cells, migration, and invasion [133]. MiR-199a interacts with the lncRNA DLX6-As1 in a FUS-dependent way to negatively influence tumor growth via invasion in CC [188]. The MAP4K1 protein upregulates the interaction of lncRNA DLX6-AS1 with miR-204-5p expression in gastric cancer (GC), promoting invasion [145–147]. DLX6-AS1 reduction inhibited tumor proliferation and MAP4K-dependent GC cell stability in an OCT1, MMP9, and SLG SLG-dependent manner. The ERK1 pathway inhibited GC cell growth by downregulating OCT1. As a result, it could be a potential therapeutic target for cancer [148]. OCT1 knock-down decreased DLX6-AS1 expression, which suppressed the expression of cell markers SLUG, MMP2, and N-cadherin [147]. As a result of these discoveries, it is possible that inhibiting lncRNA leads to therapeutic treatments for cancer progression.

The lncRNA HOTAIR interacts with miR-218, miR-545, and miR-203a-3p via a negative impacting mechanism, producing up-regulation of EGFR, β-catenin, Groucho related gene 5 (GRG5), and VOPP1 pathways, resulting in chemotherapy resistance and invasion in CRC [114, 115]. Furthermore, HOTAIR was revealed to be capable of positively modulating human epithelial growth factor receptor 2 (HER2) in GC by competing for miR-331-3p binding, a miRNA with different target specificities for both HOTAIR and HER2. When the lncRNA HOTAIR was knocked out, cell invasion and survival were reduced, but cell invasion, migration, and proliferation were boosted when HOTAIR was expressed ectopically in the GC [187].

LncRNAs bind to miR-34a and disrupt miRNA target gene expression, increasing CRC proliferation, migration, and invasion. LncRNAs such as lnc34a, which is prevalent in gastric adenocarcinoma, colon cancer stem cells (CCSCs), and short nucleolar RNA host gene 7 (SNHG7) bind to miR-34a and produce miRNA dysregulation [36, 184, 185]. Furthermore, Opa-interacting macromolecule five antisense polymer one (OIP5-AS1) interacts with miR-369e3p, leading to an increase in the expression of dual-specificity tyrosine-regulated enzyme 1 A (Dyrk1A) RNA in CRC cells [119]. OIP5-AS1 interacted adversely with miR-143-3p, inducing CC development via cellular proliferation, invasion, and metastasis [144].

Additionally, the lncRNA LIFR antisense polymer one (LIFRAS1), when interacting with miR-29a in a tumor gangrene issue (TNFAIP3) dependent manner, has a function in treatment resistance in CRC [120]. The lncRNA H19-derived from miR-675 may play an oncogenic role in gastric carcinogenesis by enhancing cell proliferation, metastasis, invasion, and migration by modifying specific miR-675 targets for poor prognosis [135, 136]. GAPLINC is another lncRNA that interacts with miR-211-3p by negatively impacting the mechanism in a CD44 oncogene-dependent manner, causing cellular proliferation and invasion [137, 138, 150]. The interaction of lncRNA HOXC6 and miR-361-3p induces upregulation, which leads to CC development [143]. The interaction of lncRNA GAS5 and miRNA 21 has been described for its role in cancer as an oncogene targeting protein-coding genes for CC advancement via invasion and metastasis via the PTEN pathway in a PDCD4 and Spry1-dependent manner [189].

## Perspectives and Expert Opinion

Cancer progression depends on extend of various factors (such as, angiogenesis, neovascularization, vascular mimicry, invasion, metastasis, epithelial-mesenchymal transition and inhibition of apoptosis) where miRNA and lncRNA interplay found critical. The inhibition of the lncRNA *via* knockdown or silencing typically inhibit the tumor progression. Therefore, the miRNA involved in the crosstalk could be utilized as a biomarker for the early detection of respective cancer. The downstream effector molecules of the signaling pathway could be used as potential therapeutic target sites for future drug discovery and utilized for tumor regression by targeting them *via* specific inhibitors. This review discussed various possibilities, where the knockdown of the lncRNA has been reported to cause tumor regression. But this method of knockdown or silencing of lncRNA for therapeutics poses a significant challenge on a large scale. In the future, we can therefore employ genome editing technologies such as ZFNs, TALENs, CRISPR to delete the necessary gene upon delivery by viral or non-viral vectors to regulate tumor progression [192, 193]. Because of the different types of cancers, tailored treatments supported by green nanotechnology could be used in clinical settings alongside mass production. We have also discussed a wide range of applications for miRNA as a diagnostic biomarker. Thus, it is important to consider conducting a clinical or pre-clinical evaluation of these biomarkers. Furthermore, only a select few scenarios are illustrated addressed in pre-clinical investigations where signals of interplay must be considered. Hence, research into signaling networks should be of interest.

## Conclusion

In conclusion, we mentioned the interaction that occurs between microRNA and long noncoding RNA, thats affects cellular functions (such as neovascularization, VM, EMT, and angiogenesis). In addition, this paper discusses a pre-clinical research on various type of malignancies encompasses the role of crosstalk between lncRNA and miRNA. This paper also focuses on developing targeting strategies for immunomodulating the cancer through cellular signaling pathways. Based on the crossplay features of lncRNAs and miRNAs, critical for cellular functions, novel cancer theranostics can be developed, such as new biomarkers or selective targeted therapy (by silencing lncRNA or knocking down miRNA levels). We extensively reviewed the essential roles of microRNA and long noncoding RNA in gene regulation so that cell-specific therapies can be developed.

## Abbreviations

AGO: Argonaute
ATG7: Autophagy Related 7
BC: Breast Cancer
CAFs: Cancer associated fibroblasts
CCAT1: Colon Cancer Associated Transcript 1
CRC: Colorectal Cancer
DLK1: Protein delta homolog 1
DLX6-AS1: Distal-less homeobox 6 antisense 1
EC: Esophageal cancer
EMT: Epithelial mesenchymal transition
FAK: Focal adhesion kinase
FOXM1: Forkhead box protein M1
FUS: Fused in sarcoma
GAPLINC: Gastric Adenocarcinoma Associated, Positive CD44 Regulator, Long Intergenic Noncoding RNA
GAS5: Growth arrest-specific
GC: Gastric cancer
GIC: Gastrointestinal Cancer
HCC: Hepatocellular Carcinoma
HDAC4: Human Histone Deacetylase 4
HGF: Hepatocyte Growth Factor
HIF-1: Hypoxia-inducible factor 1
HMGA2: High Mobility Group AT-Hook 2
HSP90: Heat shock protein 9
LC: Lung Cancer
LncRNAs: Long noncoding RNAs
LIFR: Leukemia inhibitory factor receptor
MALAT1: Metastasis Associated Lung Adenocarcinoma Transcript 1
MAP4K: Mitogen-activated protein kinase
MEG3: Maternally expressed gene 3
miRNAs: microRNAs
MMP9: Matrix metallopeptidase 9
MRE: MicroRNA Response Element
NSCLC: Non-small-cell lung carcinoma
OCT1: Octamer-binding transcription factor 1
OC: Ovarian Cancer
PC: Pancreatic Cancer
PTEN: Phosphatase and tensin homolog
SLUG: Zinc-finger transcription factor SNAI2
SOX: SRY-Box Transcription Factor
STAT3: Signal transducer and activator of transcription 3
TC: Tongue cancer
TGF: Transforming growth factor
TME: Tumor Microenvironment
UCA1: Urothelial carcinoma associated 1
UICLM: Upregulated in colorectal cancer liver metastasis
WTAPP1: Wilms tumor 1 associated protein pseudogene 1
XIST: X-inactive specific transcript
ZEB2: Zinc finger E-Box binding homeobox 2
